# *A*_2_AgCrBr_6_ (*A* = K, Rb, Cs) and Cs_2_AgCrX_6_(X = Cl, I) Double Perovskites: A Transition-Metal-Based Semiconducting Material Series with Remarkable Optics

**DOI:** 10.3390/nano10050973

**Published:** 2020-05-18

**Authors:** Pradeep R. Varadwaj

**Affiliations:** Department of Chemical System Engineering, School of Engineering, The University of Tokyo 7-3-1, Tokyo 113-8656, Japan; pradeep@t.okayama-u.ac.jp or prv.aist@gmail.com

**Keywords:** halide double perovskites, effect of chromium, stability, DOS and band structures, transport and optical properties, DFT studies

## Abstract

With an interest to quest for transition metal-based halogenated double perovskites *A*B′B″X_6_ as high performance semiconducting materials for optoelectronics, this study theoretically examined the electronic structures, stability, electronic (density of states and band structures), transport (effective masses of charge carriers), and optical properties (dielectric function and absorption coefficients, etc.) of the series *A*_2_AgCrBr_6_ (*A* = K, Rb, Cs) using SCAN + *rVV*10. Our results showed that *A*_2_AgCrBr_6_ (*A* = Rb, Cs), but not K_2_AgCrBr_6_, has a stable perovskite structure, which was revealed using various traditionally recommended geometry-based indices. Despite this reservation, all the three systems were shown to have similar band structures, density of states, and carrier effective masses of conducting holes and electrons, as well as the nature of the real and imaginary parts of their dielectric function, absorption coefficient, refractive index, and photoconductivity spectra. The small changes observed in any specific property of the series *A*_2_AgCrBr_6_ were due to the changes in the lattice properties driven by alkali substitution at the *A* site. A comparison with the corresponding properties of Cs_2_AgCrX_6_ (X = Cl, I) suggested that halogen substitution at the X-site can not only significantly shift the position of the onset of optical absorption found of the dielectric function, absorption coefficient and refractive spectra of Cs_2_AgCrCl_6_ and Cs_2_AgCrI_6_ toward the high- and low-energy infrared regions, respectively; but that it is also responsible in modifying their stability, electronic, transport, and optical absorption preferences. The large value of the high frequency dielectric constants—together with the appreciable magnitude of absorption coefficients and refractive indices, small values of effective masses of conducting electrons and holes, and the indirect nature of the bandgap transitions, among others—suggested that cubic *A*_2_AgCrBr_6_ (*A* = Rb, Cs) and Cs_2_AgCrCl_6_ may likely be a set of optoelectronic materials for subsequent experimental characterizations.

## 1. Introduction

Innovative materials that display impressive geometrical, stability, electronic, transport, and optical characteristics in the infrared through visible to ultraviolet region of the electromagnetic spectrum are a special class of semiconductors for application in optoelectronic devices [1,2,3,4,5,6,7,8,9]. Halide based single [10,11,12,13,14,15,16,17] and double perovskites [1,2,3,4,5,6,7,8,9,18,19,20,21,22] known by the chemical formulae *A*BX_3_ and *A*B′B″X_6_, respectively, are examples of such compounds. Not only do they absorb light, but many of them also emit light in specific regions of the electromagnetic spectrum, depending on the nature of ingredients at the atomic scale with which they are built. In single halide perovskites with *A*BX_3_ composition [10,11], *A* is the organic or inorganic species in the +1 oxidation state (viz. CH_3_NH_3_^+^, HC(NH_2_)_2_^+^, C(NH_2_)_3_^+^, Cs^+^, Na^+^), X is the halogen derivative in the −1 oxidation state (viz. F^−^, Cl^−^, Br^−^, I^−^), and B = transition metal (d-block) or non-transition metal (p-block) element in the +2 oxidation state (viz. Pb^2+^, Sn^2+^, and Ge^2+^). In halide double perovskites with *A*B′B″X_6_ composition [1,2,3,4,5,6,7,8,9,18,19,20,21,22,23,24], the B′ site is usually occupied with a monovalent transition metal or an alkali metal (e.g., Cu+, Ag+, and Na^+^) and the B″ site is occupied with a transition metal or non-transition metal cation in the +3 oxidation state (e.g., Cr^3+^, In^3+^, Sb^3+^, Bi^3+^).

Although *A*BX_3_ halide perovskites have been identified as best light absorbers with the largest PCE (power conversion efficiency) of 25.2% for CH_3_NH_3_PbI_3_ [25], most of the halide-based double perovskites experimentally characterized to date were recognized as weak absorbers of light in the infrared and visible regions. Cs_2_AgBiBr_6_ is one of such largely studied halide double perovskites that found application in the development of solar cells [2,3]. Vacancy-ordered *A*_2_B^4+^X_6_ halide double perovskites (viz. Cs_2_TiBr_6_) exhibit favorable bandgaps, with PCE can go up to 3.3% [4]. Whilst *A*B′B″X_6_ exhibits low PCE (viz. 2.43% for Cs_2_AgBiBr_6_) [2,3], several of them have been demonstrated to behave as photodetectors. For instance, Cs_2_AgInCl_6_ is a single crystal-based UV detector that has displayed best performance with visible blind, low dark current (~10 pA at 5 V bias), fast photoresponse (~1 ms), high ON–OFF ratio (~500), and high detectivity (~1012 Jones) [2]. Cs_2_AgBiBr_6_ thin films were also regarded as photodetectors, as they exhibit high responsivity of 7.01 A W^−1^, specific detectivity of 5.66  ×  10^11^ Jones, and ON–OFF ratio of 2.16  ×  10^4^, among other impressive properties [5]. When Cs_2_AgBiBr_6_ is embedded in a polymer matrix, it can behave as an X-ray detector [6]. Cs_2_AgBiBr_6_ nanocrystals, as a photocatalyst, were used to conduct the CO_2_ reduction reactions with high selectivity and stability. Similarly, the Bi-doped Cs_2_SnCl_6_ systems were identified as blue emissive phosphors, where Bi^3+^ is the luminescent dopant [7]. The Cs_2_(Ag_0.60_Na_0.40_)InCl_6_ system optimally alloyed with 0.04% Bi^3+^ doping was shown to emit warm white light with 86 ± 5% quantum efficiency, working for over 1000 h [8,9].

Whereas the invention of *A*B′B″X_6_ halide double perovskites has been known over decades [26,27,28,29,30,31,32], their importance as photovoltaic and luminescent materials has only recently been realized [1,2,3,4,5,6,7,8,9,18,19,20,21,22,23,24]. This came after the rationalization that lead-containing halide perovskites, such as CH_2_NH_3_PbX_3_, are environmentally unfriendly and toxic, which can be easily decomposed when exposed to UV light, heat, water pressure, ambient air oxygen, etc. [33,34,35,36]. Therefore, thousands of halide double perovskites have been investigated using high-throughput screening to discover high performance materials for photovoltaic and optoelectronic applications [37,38,39,40,41]. For instance, *A*BiCuX_6_ [A = Cs_2_, (MA)_2_, (FA)_2_, CsMA, CsFA, MAFA; X = I, Br, Cl] were theoretically shown suitable for photovoltaic and optoelectronic applications [42]. In fact, dozens of such compounds have been synthesized using experimental methods and characterized [1,2,3,4,5,6,7,8,9,18,19,20,21,22,23,24]. Many of them were found to be environmentally stable and non-toxic. This is probably because the lead ion of *A*PbX_3_ was replaced by a combination of monovalent and trivalent ions, or a tetravalent ion and a vacancy site, and that this arrangement also leads to the same overall charge balance expected of traditional halide perovskites [33,34,35,36]. As indicated above, the main problem associated with the experimentally known halogenated double perovskites is that most of them exhibited poor PCE and are not well suited for efficient solar cell designs. Therefore, experimental and theoretical studies on similar systems with different atomic compositions at the *A*, B′, and B″ sites may lead to any large scale emergence of such materials, as such substitutions would assist not only in regulating the crystal structures and defects, but also in modifying their electronic, transport, and optical properties [38,39,40,41,42,43].

We are basically interested in the theoretical modeling of *A*B′B″X_6_ systems, where the B″-site is a transition metal cation from the first and second rows of the periodic table. Substitution/doping at the B″ site by the trivalent transition metal cations (or by the non-transition metal cations) has already resulted in the synthetic development of Mn^−^ [44,45], Cu^−^ [46] and Cr-doped [23,24] Cs_2_AgInCl_6_, as well as of Yb- and Mn-doped Cs_2_AgBiX_6_ (X = Cl^−^, Br^−^) systems [47], with impressive (magneto) optoelectronics. 

This study aims to investigate the geometric, electronic, transport, and optical properties of the series *A*_2_AgCrBr_6_ (*A* = K, Rb, Cs) using density functional theory. Our main interest lies in investigating the traits of the first two heaviest members of the series, as commonly reported for similar systems with different B″-site species [1,2,3,4,5,6,7,8,9,18,19,20,21,22,23,24]. However, we extend our calculations for another lighter member of the series to deduce the extent to which the monovalent cation K at the *A* site can modify the geometric, electronic, and optical properties. The stability of the perovskite nature of *A*_2_AgCrBr_6_ is investigated using a set of geometry-based indices such as the octahedral factor [48], Goldschmidt’s tolerance factor [49,50], global instability index [51,52], and the newly proposed tolerance factor [39]. We investigate the nature of orbital contribution of each atom type leading to the formation of the valence band maximum (VBM) and conduction band minimum (CBM). In addition, we investigate the optical properties [53,54,55,56,57]—e.g., the spectra of dielectric function, absorption coefficient, electrical conductivity, complex refractive index, and the Tauc plot—and compare them with those reported for similar perovskite systems. Finally, we perform similar calculations for Cs_2_AgCrX_6_ (X = Cl, I) to elucidate the character and magnitude of bandgap transitions and the extent to which halogen substitution at the X-site modifies the geometrical, stability, transport, and optoelectronic properties of Cs_2_AgCrBr_6_.

## 2. Computational Details

For reasons discussed in various previous studies [58,59,60,61,62,63], the geometries of *A*_2_AgCrBr_6_ (*A* = Cs, Rb, K) and Cs_2_AgCrX_6_ (X = Cl, I) were relaxed with SCAN + *rVV1*0 [58]. The SCAN functional is “strongly constrained and appropriately normed”, which is paired with the non-local correlation part from the *rVV1*0 vdW density functional. It is one of the most suitable functionals recommended for the study the atomic structure of hybrid perovskite materials [63]. The k-point mesh 8 × 8 × 8 centered at Γ was used for sampling the first Brillouin zone. The projector augmented-wave (PAW) method [64] was used. The energy cut-off used for geometry relaxation was 520 eV. The cut-off in total energy for the relaxation of the electronic degrees of freedom was set to 10^−8^ eV per cell, instead of the default value of 10^−4^. A negative value of −0.01 for EDIFFG was set that defines the break condition for the ionic relaxation loop. The average forces on ions were minimized to 0.005 eV/Å. Dynamical and mechanical stabilities of the studied systems were not examined, as this may be the subject of another extensive study. All spin-polarization calculations, without considering the effect of spin-orbit coupling, were performed using VASP 5.4 [65,66]. 

The electronic band dispersions were calculated using the same functional SCAN + *rVV*10 and the same band levels (Γ(0,0,0), X(1/2,0,1/2), L(1/2,1/2,1/2), W(*k* = 1/2,1/4,3/4)) of the Fm-$\bar{3}$m space group [67] previously used for Cs_2_InAgCl_6_ [43] were utilized. The density of states (DOS) was calculated with the tetrahedron method that incorporates Blöchl corrections [68]. The Pyband [69] and Sumo [70] packages were used to plot the DOS and band structures, respectively. To shed some light on the nature of mobility of charge carriers in *A*_2_AgCrBr_6_ (*A* = Cs, Rb, K) and Cs_2_AgCrX_6_ (X = Cl, I), the effective masses of electrons and holes (m_e_^*^ and m_h_^*^, respectively) were calculated by parabolic fitting of the band edges. The relationship m*=±η2∂2E(k)∂k2 was invoked, where *E*(*k*) is the energy of the band edge and the + and – signs refer the electrons and holes, respectively [71].

The frequency-dependent dielectric function (*ε*(*ω*)) is one of the most important linear response functions often examined in the area of materials science and nanotechnology since it is the determinant of the optical properties of materials [53,54,55,56,57]. *ε*(*ω*) was calculated using Equation (1), where *ε*_1_(ω) = $\varepsilon_{\alpha \beta }^{\left(1\right)}(\omega )$ is the real part, and *ε*_2_(*ω*) = $\varepsilon_{\alpha \beta }^{\left(2\right)}(\omega )$ is the imaginary part of *ε*(*ω*).

(1) $$\varepsilon (\omega )=\varepsilon_{1}(\omega )+i\varepsilon_{1}(\omega )=\varepsilon_{\alpha \beta }^{\left(1\right)}(\omega )+i\varepsilon_{\alpha \beta }^{\left(2\right)}(\omega )$$

$\varepsilon_{\alpha \beta }^{\left(1\right)}(\omega )$ is related to $\varepsilon_{\alpha \beta }^{\left(2\right)}(\omega )$ by the Kramers-Kronig transformation [53,72] and is given by Equation (2), where *P* denotes the principal value, and η is a small complex shift.

(2) $$\varepsilon_{\alpha \beta }^{\left(1\right)}(\omega )=1+\frac{2}{\pi }P\int_{0}^{\propto }\frac{\varepsilon_{\alpha \beta }^{\left(2\right)}(\omega^{\prime })\omega^{\prime }}{{\omega^{\prime }}^{2}-\omega^{2}+i\eta }d\omega^{\prime }$$

$\varepsilon_{\alpha \beta }^{\left(2\right)}(\omega )$ was calculated using the Kubo-Greenwood formula given by Equation (3) [72], where subscripts in italics *c* and *υ* refer to conduction and valence band states, respectively, and *u_ck_* is the cell periodic part of the orbitals at the *k*-point *k*.

(3) $$\varepsilon_{\alpha \beta }^{\left(2\right)}(\omega )=\frac{4\pi^{2}e^{2}}{\Omega }\lim_{q\to 0}\frac{1}{q^{2}}\sum_{c,\upsilon ,k}2w_{k}\delta (\varepsilon_{ck}-\varepsilon_{\upsilon k}-\omega )\times \langle u_{ck+e\alpha q}|u_{\upsilon k}\rangle \langle u_{\upsilon k+e\beta q}|u_{\upsilon k}\rangle$$

The calculation of dielectric function has often been performed with dense *k*-point meshes, which is arguably because of the convergence of dielectric constant with respect the number of *k*-points leading to greater chemical accuracy [73]. Because of limited computational resources, we carried out such calculations using a 4 × 4 × 4 k-point mesh, in which, the number of empty conduction band states used was doubled and the number of grid points on which to compute DOS was set to a recommended value of 2000 [74]. Since the calculation of the linear response properties cannot be performed with the meta-GGA functional (SCAN + *rVV*10) using VASP 5.4 [65,66], we used the PBESol functional [75], in combination with the density functional perturbation theory (DFPT) method [76,77,78], to calculate such properties. The SCAN + *rVV*10 relaxed geometries of *A*_2_AgCrBr_6_ (A = Cs, Rb, K) and Cs_2_AgCrX_6_ (X = Cl, I) were used. Optical spectral properties, viz. photoabsorption coefficient *α(ω)* [55], photoconductivity σ(*ω*) [57], refractive index *n*(*ω*) [54,55,56], and extinction coefficient *κ(ω)* [54,55,56] spectra were calculated using Equations (4)–(7), respectively.

(4) $$\alpha (\omega )=\frac{\sqrt{2}\omega }{c}{\left\{{\left[\varepsilon_{1}^{2}+\varepsilon_{2}^{2}\right]}^{{}^{\frac{1}{2}}}-\varepsilon_{1}(\omega )\right\}}^{\frac{1}{2}}$$

(5) $$\sigma (\omega )=\frac{4\varepsilon_{0}E}{e}\varepsilon_{2}(\omega )$$

(6) $$n(\omega )={\left[\frac{\sqrt{\varepsilon_{1}^{2}+\varepsilon_{2}^{2}}+\varepsilon_{1}}{2}\right]}^{\frac{1}{2}}$$

(7) $$\kappa (\omega )={\left[\frac{\sqrt{\varepsilon_{1}^{2}+\varepsilon_{2}^{2}}-\varepsilon_{1}}{2}\right]}^{\frac{1}{2}}$$

In Equation (4), c and *ω* are the speed of light in vacuum and the frequency of light wave, respectively. In Equation (5), *E* is the energy of light in eV, *ε*_0_ is the permittivity of vacuum in C^2^/Nm^2^, and *e* is the charge of electron in Coulomb. The calculated values of real and imaginary parts of *ε*(*ω*) were used for the calculation of optical constants given by Equations (4)–(7) [65,66]. 

## 3. Results and Discussion

### 3.1. Geometrical Properties and Stability of Perovskite Structures

**Figure 1:**
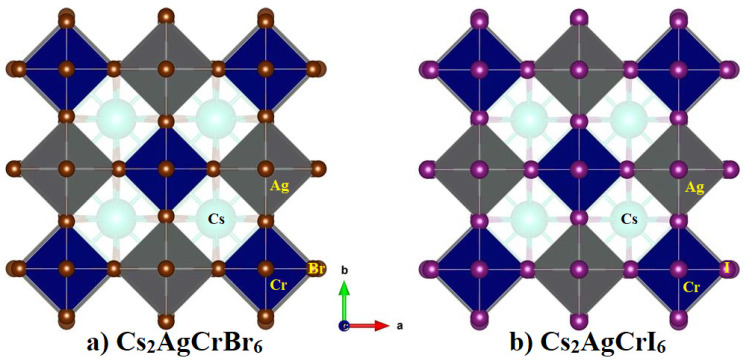
Comparison of the relaxed polyhedral model of (**a**) Cs_2_AgCrBr_6_ with (**b**) Cs_2_AgCrI_6_. Labeling of atom type is marked.

Table 1 summarizes the calculated lattice constants (*a*, *b*, *c*, α, β, and γ), cell volume (*V*) and density (ρ) of *A*_2_AgCrBr_6_ (*A* = Cs, Rb, K). Figure 1a shows the relaxed polyhedral model of Cs_2_AgCrBr_6_, which is analogous with that found for *A*_2_AgCrBr_6_ (*A* = Rb, K). Because *a* = *b* = *c* and α = β = γ for each of them, each adopts a 3D elpasolite structure with space group Fm-$\bar{3}$m. 

The lattice constant *a* is found to be the largest of 10.68 Å for Cs_2_AgCrBr_6_, and the smallest of 10.52 Å for K_2_AgCrBr_6_. The trend in the decrease of *a* in the series is consistent with the corresponding decrease in the cell volume *V*, with the *V* values ranging from 1217.10 Å^3^ (Cs_2_AgCrBr_6_) to 1165.60 Å^3^ (K_2_AgCrBr_6_). These changes are in agreement with the corresponding decrease in ρ, as well as that in the Ag–Cl, Cr–Cl, and *A*–Cl bond distances (Table 2), across the series passing from Cs_2_AgCrBr_6_ through Rb_2_AgCrBr_6_ to K_2_AgCrBr_6_. For comparison, our calculation on Cs_2_AgBiCl_6_ and Cs_2_AgBiBr_6_ yielded in lattice constants, density, and volumes that were not only close to experiment (within 2%), but also led to a suggestion that the Cr-substituted compounds are relatively lightweight, thus advocating the reliability of the accuracy of the DFT functional chosen. 

The geometrical stability of *A*_2_AgCrBr_6_ was examined using the global instability index, *GII*, given by: $GII=\sqrt{\sum_{i=1}^{n}\frac{d_{i}^{2}}{n}}$, where *n* is the number of ions and *d* is the bond discrepancy factor defined as the deviation of bond valence sum (*BVS*) from formal valence [51,52]. *BVS* was calculated using the sum of bond valences ($s_{ij}$) around any specific ion given by: $BVS=\sum_{i=1}^{n}s_{ij}$, where $s_{ij}=\exp(\frac{l_{0}-l_{ij}}{b})$, *l_ij_* is a bond length, *l*_0_ is the bond valence parameter empirically determined using experimental room-temperature structure data, and *b* is the bond softness parameter. For geometrically stable perovskite structures without steric distortions, *GII* equals 0.0 valence unit (v.u.); and for empirically unstable structures, *GII* > 0.2 v.u. [51,52]. 

The results of our calculation listed in Table 1 show that *GII* for Cs_2_AgCrBr_6_, Rb_2_AgCrBr_6_ and K_2_AgCrBr_6_ are 0.02, 0.06, and 0.08 v.u., respectively. This means that all the studied systems are associated with very marginal geometrical distortion compared to what might be expected of ideal perovskite structures. The largest instability is observed for K_2_AgCrBr_6,_ which is believed to be due to the small size of the K^+^ cation that causes the lattice K_2_AgCrBr_6_ to contract. 

Table 3 lists Shannon’s ionic radii of atoms that were used to understand the geometrical stability of *A*_2_AgCrBr_6_. Specifically, we used them for the calculation of the octahedral factor *μ* (*μ* = *r_B_*/*r_X_*), and Goldschmidt tolerance factor ($t=\frac{r_{A}+r_{X}}{\sqrt{2}(r_{B}+r_{X})}$). According to numerous previous demonstrations which have appeared in the literature [48,49,50], the combination (*μ* and *t*) should be suitable to assess the formability of perovskite structures provided *μ* and *t* values are in the ranges 0.414 < *μ* < 0.732 and 0.825 < *t* < 1.059, respectively. Our calculation gave a value of 0.45 for *μ* for all *A*_2_AgCrBr_6_. Similarly, the *t* values for these systems were in the range 0.90 < *t* < 0.96 (Table 3). Clearly, the combination (*μ* and *t*) recognizes the formability of *A*_2_AgCrBr_6_ (*A* = Cs, Rb, K) as stable perovskites. For comparison, the most studied CH_3_NH_3_PbI_3_ was reported to have a *t* of 0.91 (unstable with respect to tilting), whereas NH_4_PbI_3_ has a *t* of 0.76 [79]. For the latter case, an alternative non-perovskite structure was suggested [79].

Table 3 also includes the *τ* (a newly proposed tolerance factor [39]) values for *A*_2_AgCrBr_6_. These were calculated using the relationship given by $\tau =\frac{r_{X}}{r_{B}}-n_{A}(n_{A}-\frac{\frac{r_{A}}{r_{B}}}{\ln(\frac{r_{A}}{r_{B}})})$, where *n_A_* is the oxidation state of *A*, *r_i_* is the ionic radius of ion *i*, and *r_A_* > *r_B_* by definition. The proposal to evaluate the geometrical stability of single and double perovskites (*ABX*_3_ and *A*_2_*B*′*B*″*X*_6_, respectively) using *τ* (*τ* < 4.18) was emerged after it was being realized that the recommended ranges for *μ* and *t* do not guarantee the formation of the perovskite structure since they give a high false-positive rate (51%) in the regions of *t* (0.825 < *t* < 1.059) and *μ* (0.414 < *μ* < 0.732). In fact, *τ* was used to generalize outside of a 1034 training set of experimentally realized single and double perovskites (91% accuracy), and that its application was also useful to identify 23,314 new double perovskites ranked by their probability of being stable as perovskite [39]. Based on the criterion that *τ* < 4.18 for geometrically stable perovskites, we found that *A*_2_AgCrBr_6_ (*A* = Cs, Rb) are a set of such geometrically stable perovskites (4.04 < *τ* < 4.14), and K_2_AgCrBr_6_ (*τ* = 4.22) is a partially unstable structure (a non-perovskite!), where the ionic radius of *B* was calculated as the arithmetic mean of the ionic radii of *B*′ and *B*″. These results are not in exact agreement with that inferred from *GII* or the combination of *μ* and *t* values (see Table 1 and Table 3). This may either mean that the value of *τ* (*τ* < 4.18 [39]) recommended for identifying unknown perovskite structures may be stringent, causing the rejection of K_2_AgCrBr_6_ as a stable perovskite, or both the *GII* and the combination of *μ* and *t* mislead the stability features. 

To understand the effect of halogen substitution at the X site of *A*_2_AgCrX_6_, we have carried out similar calculations only for Cs_2_AgCrI_6_ and Cs_2_AgCrCl_6_. The calculated lattice constants, cell volume, and density were 11.48 Å, 1511.9 Å^3^, and 5.2 gcm^−3^ for Cs_2_AgCrI_6_, respectively. These were 10.13 Å, 1040.2 Å^3^, and 4.08 gcm^−3^ for Cs_2_AgCrCl_6_, respectively. In other words, the halogen substitution does not cause significant strain in the lattice that can lead to the change in the symmetry of the system from Fm-$\bar{3}$m. However, the replacement of the Br atoms in Cs_2_AgCrBr_6_ by the Cl and I anions has indeed resulted in lattice contraction and expansion, respectively, which are expected to affect the electronic, transport, and optical properties of the resulting systems (*vide infra*). The presence of lattice contraction and expansion is also evident of metal-halide and alkali-halide bond lengths that are decreasing across the series in this order: Cs_2_AgCrCl_6_ < Cs_2_AgCrBr_6_ > Cs_2_AgCrI_6_. (A comparison detail of the Ag–X, Cr–X, and Cs–X bond distances for Cs_2_AgCrX_6_ (X = Cl, Br, I) is given in Table 2).

Moreover, the calculated *μ* and *t* values were 0.40 and 0.94 for Cs_2_AgCrI_6_ (Table 3), respectively. Based on the traditional arguments [48,50], one may not disapprove to call Cs_2_AgCrI_6_ a stable perovskite. The same argument applies to Cs_2_AgCrCl_6_, as the combination of the *μ* and *t* values (Table 3) favors a perovskite structure. This result is consistent with that inferred from the *GII* values (0.10 v.u. for Cs_2_AgCrCl_6_ and 0.00 v.u. for Cs_2_AgCrI_6_). However, our calculated *τ* value of 4.31, far exceeding its recommended value of 4.18, rejects Cs_2_AgCrI_6_ as a stable perovskite. This is analogously as *τ* rejected the K_2_AgCrBr_6_ system to be called a perovskite. However, this is not the case with Cs_2_AgCrCl_6_ (*τ* = 3.87), in agreement with that reported recently [39]. Clearly, the aforesaid mismatch between the results associated with different indices suggests that the geometrical properties of a large body of *A*B′B″X_6_ systems of different B′ and B″compositions, together with X = I and Br and *A* = K, need to be analyzed to reach any definitive conclusion on the predictability of *GII-* and *τ*-based stability features.

### 3.2. Density of States and Band Structures

**Figure 2:**
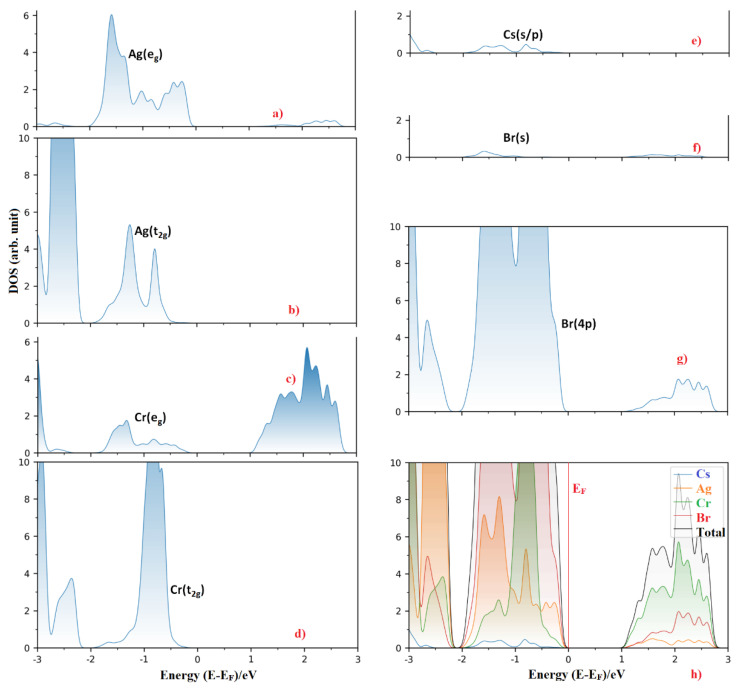
(**a**–**g**) The orbital-projected partial density of states of selective atom type for Cs_2_AgCrBr_6_. Included in (**h**) is the atom-projected density of states for the corresponding system. The Fermi level E_F_ is marked at 0 eV in (h).

Figure 2a–g shows the orbital-projected partial density of states of each atom type, and Figure 2h shows the atom projected partial density of states for Cs_2_AgCrBr_6_. From these, it may be said that the dispersion of the HOMO band (VBM) results collectively from contributions of e_g_ and t_2g_ states of both Ag (4d) and Cr(3d), and the 4p states of Br. This is also reminiscent of the data provided in Table 4, in which, the VBM is mainly due to the 4p states of Br (88%), and that the contribution from the 3d (Cr) and 4d (Ag) states is very small. 

**Figure 3:**
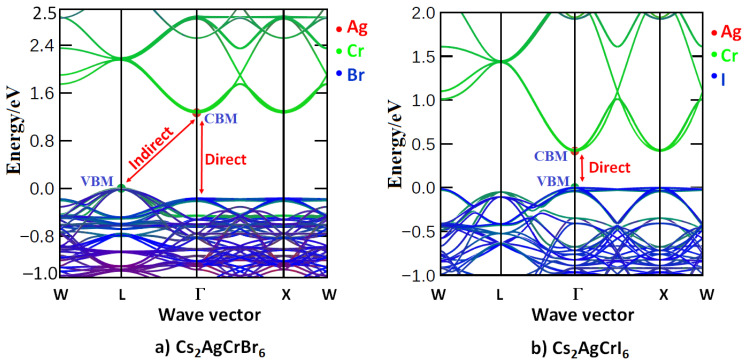
Comparison of the electronic band dispersion of (**a**) Cs_2_AgCrBr_6_ with (**b**) Cs_2_AgCrI_6_. The nature of band dispersion shown in (**a**) is virtually identical with those of *A*_2_AgCrBr_6_ (*A* = Rb, K) (not shown). The indirect nature of the bandgap with the VBM at the L- and Γ-points in (**a**) are also marked and Table 1 lists the E_g_ values for Cs_2_AgCrBr_6_. The conventional unit-cell geometries were used to calculate the band dispersion.

**Figure 4:**
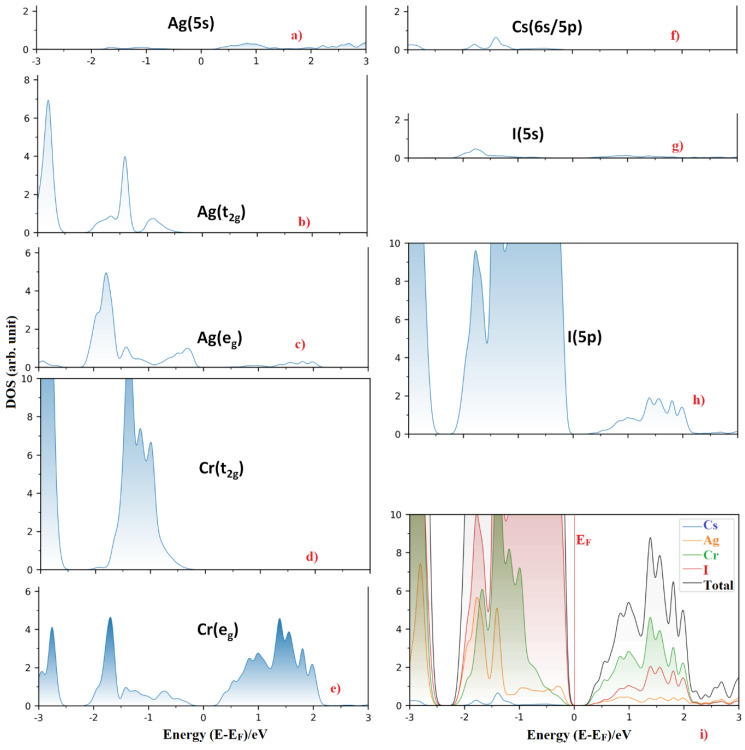
(**a**–**h**) The orbital-projected partial density of states of selected atom type for Cs_2_AgCrI_6_. Included in (**i**) is the atom-projected density of states for the corresponding system. The Fermi level E_F_ is marked at 0.0 eV.

On the other hand, the LUMO band (CBM) of Cs_2_AgCrBr_6_ is predominantly of Cr (3d) character. It is substantially composed of the e_g_ orbital energy states (~72.5%). This, together with the small contributions from the Ag (5s) and Br (4p) states, causes the dispersion of the band. Note that alkali substitution at the *A* site has very little effect on the orbital characters of VBM and CBM. However, when the Br atom at the X-site was replaced by the I and Cl atoms, this had a very marginal effect on the orbital character of the CBM, and significant effect on the VBM. In particular, the 3d (Cr) energy states dominate below the Fermi level for Cs_2_AgCrCl_6_ (46%), which was comparatively larger than those arose from the 3p (35.2%) and 4d (17.0%) states of Cl and Ag, respectively. For Cs_2_AgCrI_6_, the I(5p) states dominate below the Fermi level, and the 3d (Cr) and 4d (Ag) states contribute marginally to the VBM. Figure 3 compares the nature of band dispersion of Cs_2_AgCrBr_6_ with Cs_2_AgCrI_6_, and Figure 4 illustrates the orbital- and atom-projected partial density of states for Cs_2_AgCrI_6_. 

From the bandgap (E_g_) data listed in Table 1, it is apparent that the *A*_2_AgCrBr_6_ systems are an indirect bandgap material. This is arguably because the VBM is located at the high symmetry L-valley (VBM) and the CBM is located at the Γ-valley (CBM); see Figure 3a for Cs_2_AgCrBr_6_. The calculated bandgaps (E_g_) of *A*_2_AgCrBr_6_ were found between 1.27 and 1.29 eV (Table 1), thereby suggesting the semiconducting nature of these materials. Small variations in E_g_ across the alkali series indicate that alkali substitution at the *A*-site of *A*_2_AgCrCl_6_ does not significantly affect the gap between CBM and VBM, and is consistent with the nature and extent of orbital characters that were involved in the formation of these bands (Table 4).

By contrast, Cs_2_AgCrI_6_ has a bandgap of 0.43 eV, and is direct at the Γ-valley (Figure 3b). Obviously, the replacement of the Br atoms of Cs_2_AgCrBr_6_ with iodine atoms not only causes the shift of the VBM from the L-valley to appear at the Γ-valley, but also significantly narrows the gap between the CBM and VBM and alters the character of the electronic transition between them. This is not the case with Cs_2_AgCrCl_6_ since the bandgap for this system is increased giving rise to an E_g_ value of 1.84 eV (SCAN + *rVV*10) and was indirect between the L-valley (VBM) and Γ–valley (CBM). These results lead to a conclusion that halogen substitution from Cl through Br to I at the X-site of Cs_2_AgCrX_6_ not only triggers the nature of the bandgap from indirect to direct, but also adjusts its magnitude from 1.84 eV to 0.43 eV. 

In any case, the charge transport properties of any semiconducting material are strongly dependent on the effective masses of the charge carriers [80,81,82]. Table 5 summarizes the effective masses of conduction electrons and holes for *A*_2_AgCrBr_6_, which were calculated by parabolic fitting of the lower conduction band and the upper valence band centered at the Γ- and L-valleys, respectively. Since the HOMO band is formed by several spin-up and spin-down channels (total eight) caused by the Cr^3+^ ions, there are spin-up and spin-down holes along the crystallographic directions. The most important of these is the L→Γ direction, which is linked directly with the electronic transition between the VBM and CBM. As can be seen from Table 5, the effective masses of the spin-up holes (m_h_*(*up*)) are always lighter than that of the spin-down holes (m_e_*(*down*)), regardless of the nature of the *A*-site in *A*_2_AgCrBr_6_. This means that (m_h_*(*down*)) would play an insignificant role in determining the transport phenomena that are usually governed by the band structures at and around the close vicinity of the Γ-point. In contrary, there are only spin-up electrons that are associated with the bottom of the conduction band. They are indeed small (m_e_*(*up*) (values lie between 0.50 and 0.62 *m*_0_ for all directions), but are not always heavier than those of m_h_*(*up*). These results suggest that the studied systems might be suitable as hole (and electron) transporting materials due to their high mobility [81,82,83]. A similar result was found for Cs_2_AgCrCl_6_ (values not given).

Table 6 collects the m_e_*(*up*) and m_h_*(*up*) values for Cs_2_AgCrI_6_. Of the three, two channels associated with the top of the valence band are having significantly smaller m_h_*(*up*) values along both the crystallographic directions (Γ→L and Γ→X) compared to that of the third one. m_e_*(*up*) is always smaller than m_h_*(*up*) regardless of the nature of crystallographic directions, which is obviously due to the ‘flatter’ nature of the VBM compared to the parabolic CBM (Figure 3b). 

For comparison, Wang et al. have reported unusually heavier effective masses for holes for the hybrid perovskite series, AEQTBX_4_ (B = Pb, Sn; X = Cl, Br, I; AEQT= H_3_NC_2_H_4_C_16_H_8_S_4_C_2_H_4_NH_3_^2+^), with the m_h_* values between 0.63 *m*_0_ ((AEQT)SnI_4_) and 105.21 *m*_0_ ((AEQT)PbI_4_) [84]. (AEQT)SnI_4_ was observed to exhibit dispersive VBM and CBM, and was accompanied by a moderate fundamental bandgap of 2.06 eV involving a strong direct valence band to conduction band transition. This, together with the relatively light effective masses for electrons and holes (~0.6 *m*_0_), and high dielectric constants, has led to a suggestion that this system is suitable for application as a top absorber of the tandem solar cell.

### 3.3. Optical Properties

**Figure 5:**
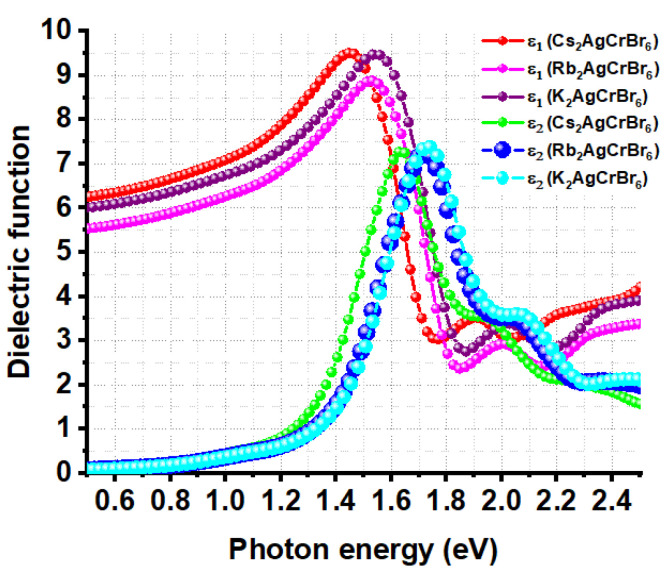
Illustration of the dependence of the real and imaginary parts (*ε*_1_ and *ε*_2_, respectively) of the dielectric function *ε* on the photon energy for *A*_2_AgCrBr_6_ (*A* = Cs, Rb, K) double perovskites.

The excitonic effect is generally approximated by solving the Bethe–Salpeter equation for the two-body Green’s function, without or with considering local field effect [85]. Due to the high computational cost and limited computed resources, the frequency-dependent complex dielectric function was calculated within the framework of DFPT without taking into account the electron-hole coupling effect. Studies have shown that neglecting electron-hole coupling can yield reasonable results for semiconductors with small band gaps [86,87]. Nevertheless, Figure 5 shows the plot of the real and imaginary parts of the dielectric function as a function of photon energy for *A*_2_AgCrBr_6_. The first peak of the *ε*_2_ curve is located at an energy higher than that of the *ε*_1_ curve, and alkali substitution at the *A*-site causes a slight fluctuation in the high frequency dielectric behavior. For instance, these peaks on the *ε*_1_ and *ε*_2_ curves are positioned at energies of 1.45 and 1.63 eV for Cs_2_AgCrBr_6_, respectively. These are 1.53 and 1.72 eV for Rb_2_AgCrBr_6_, respectively, and are 1.55 and 1.74 eV for K_2_AgCrBr_6_, respectively. Similarly, the low frequency limit of the isotropically averaged value of *ε*_1_ (*ω* = 0) = *ε*_∞_ (called optical dielectric constant or high-frequency dielectric constant) is found to be 6.1 for Cs_2_AgCrBr_6_, 5.4 for K_2_AgCrBr_6_, and 5.8 for K_2_AgCrBr_6_. This result indicates that the contribution of electrons to the static dielectric constant is appreciable and that the studied systems contain ionic bonds. Zakutayev et al. [88], as well as others [84], have previously demonstrated that perovskites with appreciable dielectric constants are defect tolerant. For comparison, MAPbI_3_ (X = Cl, Br, I), and other perovskite systems [73] were reported to have virtually similar optical dielectric constants (viz. *ε*_∞_ = 6.5 for MAPbI_3_, 5.2 for MAPbBr_3_, 4.2 for MAPbCl_3_, and 5.3 for CsPbI_3_).

The magnitude of the bandgap is the determinant of the onset of first optical absorption, which can be approximated using the spectra of *ε*_2_. As such, the onset of absorption is approximately lying between 1.15 and 1.40 eV for *A*_2_AgCrBr_6_ (Figure 5), showing an appreciable absorption mostly in the infrared spectral region. Note that the bandgaps of *A*_2_AgCrBr_6_ evaluated using SCAN + *rVV*10 were lying between 1.27 and 1.29 eV, and the dielectric functions were calculated using PBEsol in conjunction with DFPT, yet there is a close match between bandgap and onset of optical absorption evaluated using the two different theoretical approaches employed. 

**Figure 6:**
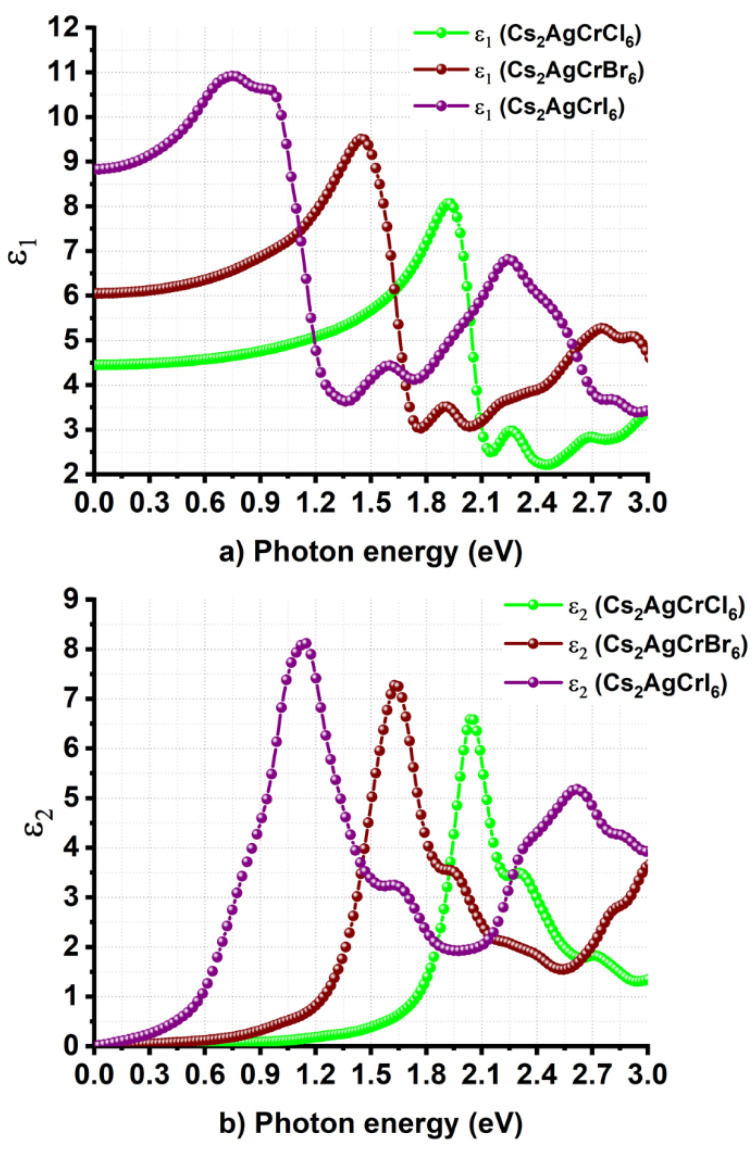
Nature of dependence of the (**a**) real and (**b**) imaginary part of dielectric function on the photon energy for Cs_2_AgCrX_6_ (X = Cl, Br, I).

Figure 6a,b compares the energy dependence of *ε*_1_ and *ε*_2_ for Cs_2_AgCrX_6_ (X = Cl, Br, I), respectively. From either of the two plots, it is apparent that halogen replacement has a significant effect not only on the magnitude of *ε*_∞_, but also on the onset of optical absorption. For instance, the value of *ε*_∞_ inferred from Figure 6a is approximately around 4.5, 6.1, and 8.9 for Cs_2_AgCrCl_6_, Cs_2_AgCrBr_6_, and Cs_2_AgCrI_6_, respectively. These indicate that Cs_2_AgCrI_6_ has a greater ability to screen charged defects compared to Cs_2_AgCrBr_6_ and Cs_2_AgCrCl_6_ [89,90]. The observed trend in the increase of *ε*_∞_ caused by halogen substitution at the X-site in Cs_2_AgCrX_6_ is consistent with that reported for the MAPbX_3_ (X = Cl, Br, I) series [73].

The onset of optical absorption corresponding to the *ε*_2_ spectra is positioned around 1.55 eV for Cs_2_AgCrCl_6_. This is significantly shifted from the near-infrared region to the low energy infrared region of the electromagnetic spectrum caused by the halogen substitution at the X-site, thus showing up at energies around 1.15 eV and 0.45 eV for Cs_2_AgCrBr_6_ and Cs_2_AgCrI_6_, respectively. These values are close to their corresponding bandgaps predicted using the SCAN + *rVV*10 functional. It is worth noting that we did not calculate the contribution of dielectric constant due to ions to the static dielectric constant, thus it is not possible to provide views on the polarity of the chemical bonds and the softness of the vibrations [91], as well as the nature and importance of (picosecond) response of lattice vibrations (phonon modes) that explain the ionic and lattice polarizations, and the extent to which the material would control the photovoltaic performance [74,89,90,92].

In any case, the major peaks in the *ε*_2_ curve of Cs_2_AgCrCl_6_ are positioned at energies of 2.0 and 2.3 eV, whereas the onset of the optical absorption was located at an energy of 1.55 eV (Figure 6b). These peaks in the UV/Vis/NIR diffuse reflectance spectrum were experimentally observed at energies of 1.6 and 2.2 eV for hexagonal Cs_2_AgCrCl_6_, respectively, and were assigned to the d-d transitions of ^4^*A*_2_→ ^4^*T*_2_ and ^4^*T*_2_→ ^4^*T*_1_, respectively. This is not surprising given that the ground state of the Cr^3+^ (t_2g_^3^e_g_^0^) cation in an octahedral O_h_ symmetry is described by ^4^*A*_2_*_g_*(*F*), and is accompanied with spin-allowed transitions from the ^4^*A*_2_*_g_*(*F*) ground state to the excited ^4^*T*_2_ and ^4^*T*_1_ states (that is, ^4^*T*_2_→ ^4^*T*_1_ and ^4^*A*_2_→ ^4^*T_1_*). Such d–d transitions were reported to occur at energies of 3.06 eV (405 nm) and 2.25 eV (550 nm) in the UV–vis spectrum of isolated Cr^3+^ complex of aspartic acid, respectively [93]. The intense and broad bands observed at energies of 2.93 eV and 2.13 eV for strontium formate dehydrate crystal of Cr^3+^ were attributed to the corresponding transitions, respectively [94]. Also, zinc-tellurite glasses doped with Cr^3+^ ion feature two intense broad bands with maxima at 454 nm (2.73 eV) and 650 nm (1.91 eV), which were assigned to the transitions ^4^*A*_2_ → ^4^*T*_1_ and ^4^*A*_2_ → ^4^*T*_2_, respectively [95]. In Cs_2_AgInCl_6_:Cr^3+^ halide double perovskite, the absorptions peaks at 1.55 eV (800 nm) and 2.19 eV (565 nm) were assigned to the same spin-allowed transitions ^4^A_2_ → ^4^T_2_ and ^4^*T*_2_→ ^4^*T*_1_, respectively. Because the VBM is dominated with Br(4p) and I(5p) energy states for Cs_2_AgCrBr_6_ and Cs_2_AgCrI_6_, respectively, and the contribution of Cr(3d) orbitals states to the VBM is reasonably small, the two electronic transitions appear in the *ε*_2_ spectra in the region 0.3–2.2 eV were shifted toward the low energy regions (Figure 6b).

**Figure 7:**
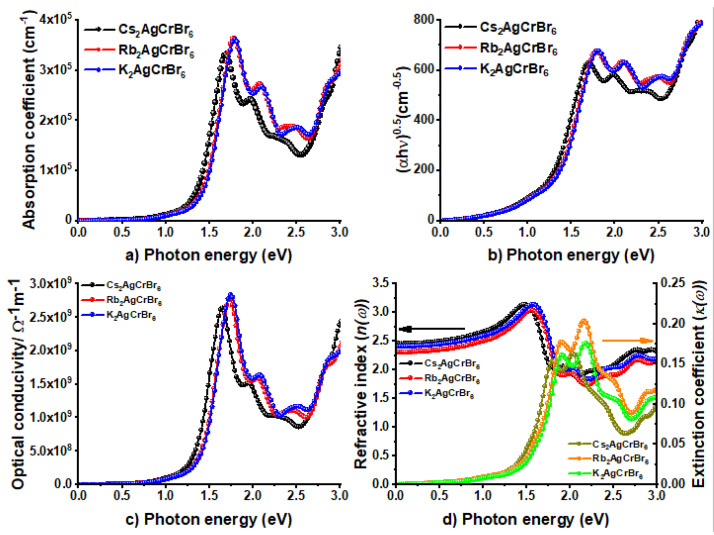
Illustration of the dependence of (**a**) absorption coefficient (*α(ω)*), (**b**) Tauc plot, (**c**) optical conductivity (*σ(ω)*), and (**d**) the real and imaginary parts of refractive index (*n* and *κ*, respectively) on the photon energy for *A*_2_AgCrBr_6_ (*A* = Cs, Rb, K).

Figure 7 displays the plot of the absorption coefficient, photoconductivity, and complex refractive index for *A*_2_AgCrBr_6_. Equations (4)–(7) were used, respectively. Figure 7b shows the Tauc plot. These plots replicate the oscillator peaks of the dielectric function *ε*_2_ in the region 0.0–3.0 eV (Figure 6). From Figure 7a, it is obvious that α for *A*_2_AgCrBr_6_, which determines the extent to which light of a particular energy can penetrate a material before it is absorbed [96], is non-zero (positive) both in the infrared and visible regions. As can be seen, different regions have different absorption coefficients. This may not be unusual given that the absorption coefficients of MAPbI_3_ as low as 10^–14^ cm^–1^ were detected at room temperature for long wavelengths, and were 14 orders of magnitude lower than those observed at shorter wavelengths [97]. Nevertheless, α increases as the light energy increases and becomes a maximum at the strongest peak occurred at an energy around 1.7 eV for Cs_2_AgCrBr_6_. The trend in α in the series *A*_2_AgCrBr_6_ follows the order: Cs_2_AgCrBr_6_ (3.3 × 10^5^ cm^−1^) < Rb_2_AgCrBr_6_ (3.6 × 10^5^ cm^−1^) ≈ K_2_AgCrBr_6_ (3.6 × 10^5^ cm^−1^). These are somehow larger than those of 0.5 × 10^4^ cm^−1^ and 1.5 × 10^4^ cm^−1^ observed at 1.77 eV (700 nm) and 2.25 eV (550 nm) for MAPbI_3_, respectively [98,99].

The nature of the curves of α is similar to that found for σ at the strongest peak positions in the region 0.0–2.5 eV (see Figure 7a vs. Figure 7c). The value of σ at these peaks is 2.64 × 10^9^ Sm^−1^ for Cs_2_AgCrBr_6_, 2.76 × 10^9^ Sm^−1^ for Rb_2_AgCrBr_6_, and 2.84 × 10^9^ Sm^−1^ for K_2_AgCrBr_6_. For comparison, the average value of σ for MAPbI_3_ thin films was reported to be 6 × 10^5^ Sm^−1^ [100].

The above trend both in α and σ is not exactly similar to that found for the static refractive index *n (ω =* 0), with the latter were around 2.5, 2.3, and 2.4 for Cs_2_AgCrBr_6_, Rb_2_AgCrBr_6_, and K_2_AgCrBr_6_, respectively (Figure 7d). The *n*(ω) values increase with respect to the increase of photon energy in the region 0.0–3.0 eV. The maximum of *n* at the highest peak in the region 0.0–3.0 eV varies between 3.0 and 3.2 for *A*_2_AgCrBr_6_.

**Figure 8:**
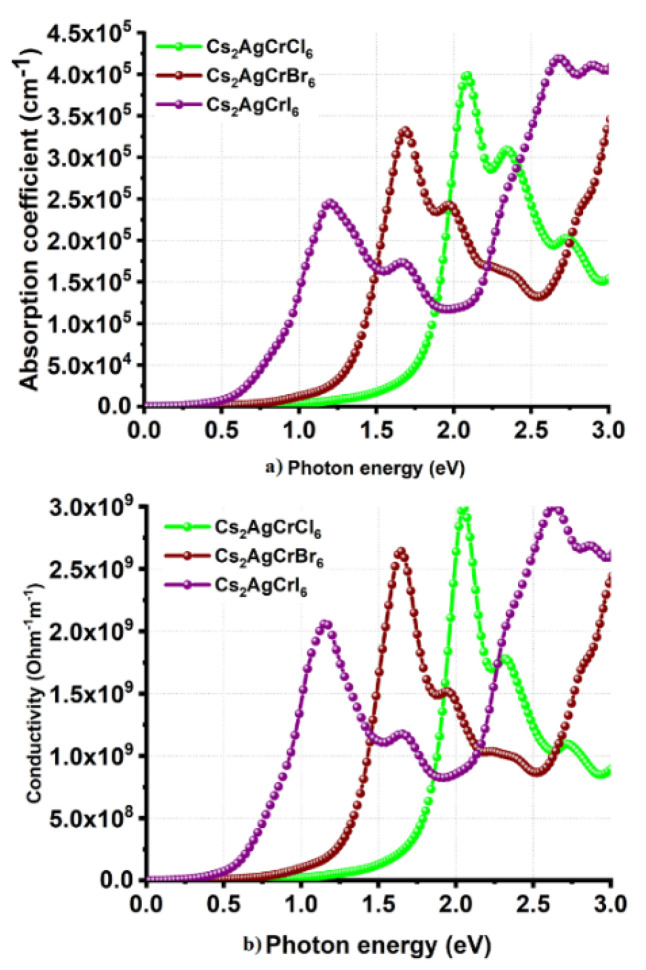
Behavior of the (**a**) absorption coefficient and (**b**) photoconductivity against photon energy for Cs_2_AgCrX_6_ (X = Cl, Br, I).

**Figure 9:**
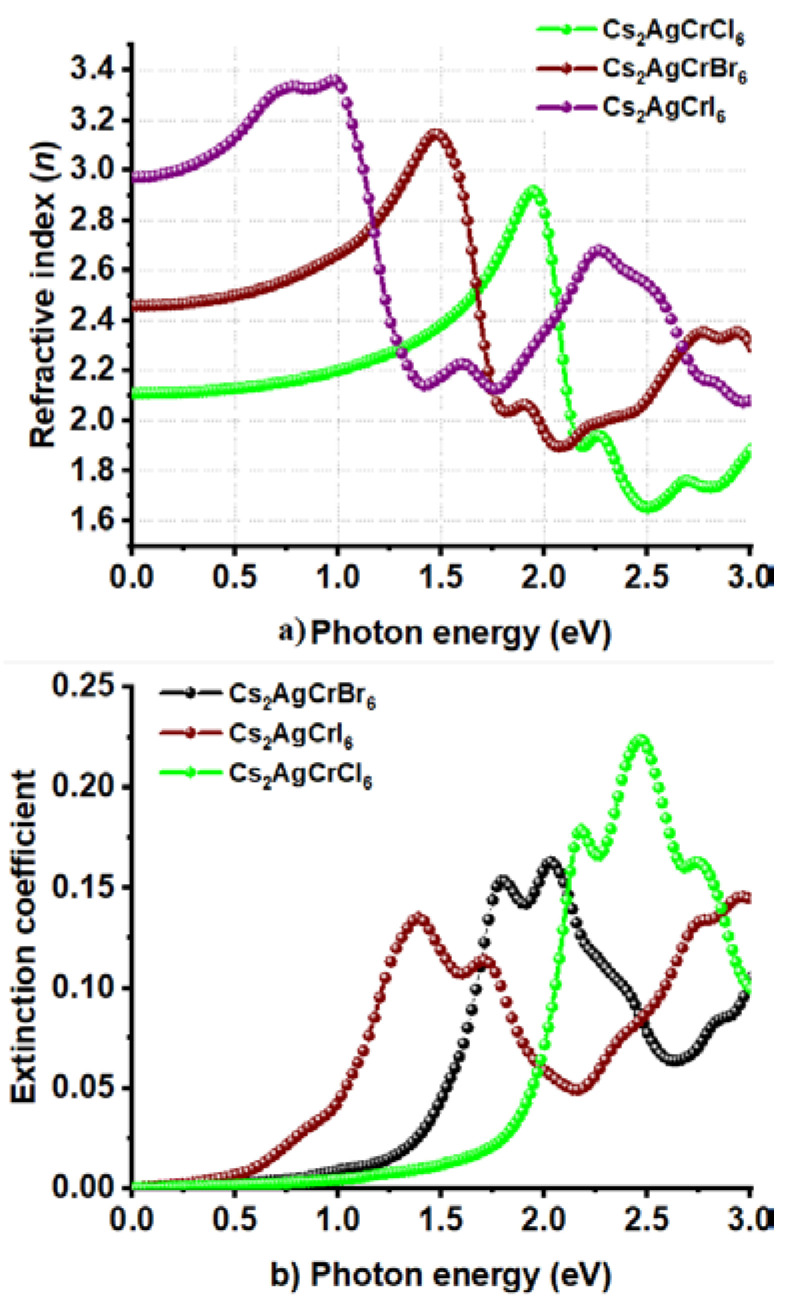
Behavior of the (**a**) real and (**b**) imaginary part of complex refractive index against the photon energy for Cs_2_AgCrX_6_ (X = Cl, Br, I).

Cl and I substitutions at the X-site in Cs_2_AgCrX_6_ has resulted in a relatively smaller α of 2.4 × 10^5^ cm^−1^ for Cs_2_AgCrI_6_ and a relatively larger α of 4.0 × 10^5^ cm^−1^ for Cs_2_AgCrCl_6_; all within the range 0.0–2.5 eV (Figure 8a). Although the peaks in the α spectrum of Cs_2_AgCrCl_6_ are relatively sharper than those of Cs_2_AgCrBr_6_ and Cs_2_AgCrI_6_, the different absorption edges found for the three systems are consistent with the corresponding bandgap properties. Also, the trend in α is concordant with the conductivity spectra shown in Figure 8b, in which, σ decreases in the series in this order: Cs_2_AgCrI_6_ < Cs_2_AgCrBr_6_ < Cs_2_AgCrCl_6_. On the other hand, the *n* (*ω* = 0) values were 2.1, 2.5, and 3.0 for Cs_2_AgCrCl_6_, Cs_2_AgCrBr_6_, and Cs_2_AgCrI_6_, respectively (Figure 9a). The maximum values of *n* were approximately 2.9, 3.2, and 3.4 for the corresponding systems, respectively, a feature which is consistent with the trend in the optical dielectric constants (see above). Regardless of the nature of the *A*_2_AgCrX_6_ systems examined, the extinction coefficient (imaginary part of the refractive index) was found to be very small (Figure 7d and Figure 9b), and is expected for semiconducting materials [101]. These results lead to a meaning that the studied Cs_2_AgCrX_6_ (X = Cl, Br, I) perovskites may result in relatively larger reflection at the perovskite/electrode interface. For comparison, the refractive indices of CH_3_NH_3_PbI_3__–__x_Cl_x_ perovskite thin films were reported approximately to be 2.4 and 2.6 in the visible to near-infrared wavelength region [101]. He et al. have examined bulk single crystals of CH_3_NH_3_PbX_3_ (CH_3_NH_3_ = MA, X = Cl, Br, I) and have measured refractive indices that rank in this order: MAPbI_3_ > MAPbBr_3_ > MAPbCl_3_ at the same wavelength [102]. These results indicate that *n* may play an important role in determining the optical response properties of halide perovskites, which may serve as a useful metric in the parameter space for use in optoelectronic device design.

## 4. Conclusions

In this study, a promising van der Waals functional called SCAN + *rVV*10 was used to theoretically investigate the electronic structures, stability, electronic, transport, and optical properties of *A*_2_AgCrBr_6_ (*A* = K, Rb, Cs). To shed some light on the effect of halogen substitution (X = Cl, I) that replaces Br in *A*_2_AgCrBr_6_ and for comparison, the same properties of Cs_2_AgCrX_6_ (X = Cl, I) obtained with the same functional were included. As such, *A*_2_AgCrBr_6_ (*A* = K, Rb, Cs) and Cs_2_AgCrX_6_ (X = Cl, I) were shown to exhibit a perovskite structure, emerged using the combined application of the octahedral factor, the Goldschmidt tolerance factor, and the global instability index. However, the application of the newly proposed tolerance factor *τ* has rejected Cs_2_AgCrI_6_ and K_2_AgCrBr_6_ as stable perovskites, showing its high-level predictability over the traditional ones. Despite the aforementioned rejection, each member of the series *A*_2_AgCrBr_6_ was shown to feature very similar bandgap properties with others, including the nature of the effective masses of charge carriers, and optical constants. Small variation in either of these properties in a given series was due to the small change in the lattice constants, cell volumes, and bonding interactions induced by alkali substitution at the *A*-site. The positional occurrence of the first onset of absorption which appeared in the spectra of the dielectric function, absorption coefficient, refractive index, and photoconductivity, as well as that in the Tauc plot, has demonstrated that the geometrically stable *A*_2_AgCrBr_6_ double perovskites emanated from this study could be interesting for their eventual experimental characterizations. We have also shown that the onset of the first absorption can be significantly shifted to the high- and low-energy infrared regions via halogen substitution at the X site with X = Cl and X = I in Cs_2_AgCrX_6_, respectively. Moreover, the computed electronic transitions of Cs_2_AgCrCl_6_ were shown in decent agreement with those reported experimentally for hexagonal Cs_2_AgCrCl_6_ and Cr-doped Cs_2_AgInCl_6_, meaning that the chosen computational model utilized in this study should be applicable to similar other halide double perovskites to explore similar properties. Because *A*_2_AgCrBr_6_ (*A* = Rb, Cs) and Cs_2_AgCrCl_6_ possess impressive electronic and optical properties, they are expected to find application in optoelectronics.

## Figures and Tables

**Table 1:** Selected lattice properties (lattice constants, volume, and density) and bandgaps of *A*_2_AgCrBr_6_ (*A* = K, Rb, Cs).

Compound	*a* = *b* = *c*/Å	α = β = γ/Deg	Volume (*V*)/Å^3^	ρ/gcm^−3^	*GII/*v.u.	E_g_/eV	Nature of E_g_	E_g_/eV	Nature of E_g_
Cs_2_AgCrBr_6_	10.68	90	1217.10	4.94	0.02	1.27	Indirect at L→Γ	1.46	Direct at Γ
Rb_2_AgCrBr_6_	10.58	90	1182.56	4.55	0.06	1.28	Indirect at L→Γ	1.50	Direct at Γ
K_2_AgCrBr_6_	10.52	90	1165.60	4.09	0.08	1.29	Indirect at L→Γ	1.52	Direct at Γ

**Table 2:** Comparison of selected bond distances *r* of *A*_2_AgCrBr_6_ (*A* = K, Rb, Cs) with Cs_2_AgCrX_6_ (X = Cl, I).

System	Bond Distances/Å		
	*r*(Ag–Br/I/Cl)	*r*(Cr–Br/I/Cl)	*r*(*A*–Br/I/Cl)
Cs_2_AgCrBr_6_	2.811	2.527	3.778
Rb_2_AgCrBr_6_	2.774	2.514	3.741
K_2_AgCrBr_6_	2.755	2.507	3.723
Cs_2_AgCrI_6_	2.977	2.761	4.059
Cs_2_AgCrCl_6_	2.697	2.369	3.586

**Table 3:** Shannon’s radii (*r*) of ions, octahedral factor (*μ*), Goldschmidt tolerance factor (*t*), and new tolerance factor (*τ*) *for A*_2_AgCrBr_6_ (*A* = Cs, Rb, K). The corresponding properties for Cs_2_AgCrX_6_ (X = Cl, I) are also included.

System	*r_A_*/Å	*r*[Ag^+^]/Å	*r*[Cr^3+^]/Å	*r_B_* = [*r*(Ag^+^) + *r*(Cr^3+^)]/2/Å	Br^–^/Å	*μ* *= r_B_/ r_X_*	*t*	*τ*
Cs_2_AgCrBr_6_	1.88	1.15	0.615	0.8825	1.96	0.45	0.96	4.04
Rb_2_AgCrBr_6_	1.72	1.15	0.615	0.8825	1.96	0.45	0.92	4.14
K_2_AgCrBr_6_	1.64	1.15	0.615	0.8825	1.96	0.45	0.90	4.22
Cs_2_AgCrCl_6_	1.88	1.15	0.615	0.8825	1.81	0.49	0.97	3.87
Cs_2_AgCrI_6_	1.88	1.15	0.615	0.8825	2.00	0.40	0.94	4.31

**Table 4:** Normalized orbital contribution of selective atoms leading to the formation of VBM and CBM of *A*_2_AgCrBr_6_ (*A* = K, Rb, Cs). Given are also the corresponding contributions responsible for the band edges of Cs_2_AgCrI_6_ and Cs_2_AgCrCl_6_. Values in %.

	VBM			CBM		
	Cr	Ag	Br/I/Cl	Cr	Ag	Br/I/Cl
	3d	4d	4p/5p/3p	3d	5s	4p/5p/3p
Cs_2_AgCrBr_6_	0.7	10.1	87.8	72.5	9.1	10.9
Rb_2_AgCrBr_6_	0.8	10.8	87.1	72.5	8.9	10.6
K_2_AgCrBr_6_	0.9	11.1	86.6	72.4	8.8	10.5
Cs_2_AgCrI_6_	7.95	2.82	86.8	69.9	8.9	12.7
Cs_2_AgCrCl_6_	46.0	17.0	35.2	74.9	10.1	9.4

**Table 5:** Effective masses of charge carriers (holes and electrons) for *A*_2_AgCrBr_6_ (*A* = Cs, Rb, K) ^a^.

Compound	Property	Crystallographic Directions															
		L→W	L→Γ	L→W	L→Γ	L→W	L→Γ	L→W	L→Γ	L→W	L→Γ	L→W	L→Γ	L→W	L→Γ	L→W	L→Γ
Cs_2_AgCrBr_6_	m_h_*/m_0_ (*up*)	−0.33	−0.47	−0.33	−0.47	−0.47	−0.47	−0.47	−0.57	−0.91	−0.56	−0.91	−0.57	−1.23	−0.84	−1.23	−0.85
	m_h_*/m_0_ (*down*)	−0.33	−3.79	−0.33	−4.23	−0.47	−4.23	−0.47	−5.14	−0.91	−4.49	−0.91	−4.79	−1.23	−5.99	−1.23	−7.19
		Γ→L	Γ→X														
	m_e_*/m_0_	0.50	0.59														
		L→W	L→Γ	L→W	L→Γ	L→W	L→Γ	L→W	L→Γ	L→W	L→Γ	L→W	L→Γ	L→W	L→Γ	L→W	L→Γ
Rb_2_AgCrBr_6_	m_h_*/m_0_ (*up*)	−0.32	−0.45	−0.32	−0.46	−0.43	−0.46	−0.43	−0.54	−0.87	−0.53	−0.87	−0.53	−1.10	−0.73	−1.10	−0.73
	m_h_*/m_0_ (*down*)	−0.32	−3.86	−0.32	−4.07	−0.43	−4.07	−0.43	−4.89	−0.87	−4.07	−0.87	−4.31	−1.10	−4.89	−1.10	−6.11
		Γ→L	Γ→X														
	m_e_*/m_0_	0.50	0.60														
		L→W	L→Γ	L→W	L→Γ	L→W	L→Γ	L→W	L→Γ	L→W	L→Γ	L→W	L→Γ	L→W	L→Γ	L→W	L→Γ
K_2_AgCrBr_6_	m_h_*/m_0_ (*up*)	−0.32	−0.45	−0.32	−0.45	−0.41	−0.45	−0.41	−0.52	−0.85	−0.52	−0.85	−0.52	−1.06	−0.68	−1.06	−0.68
	m_h_*/m_0_ (*down*)	−0.32	−3.70	−0.32	−3.90	−0.41	−4.11	−0.41	−4.63	−0.85	−4.11	−0.85	−4.11	−1.06	−4.63	−1.06	−5.69
		Γ→L	Γ→X														
	m_e_*/m_0_	0.50	0.61														

^a^m_h_* and m_e_* in units of electron rest mass *m*_0_ (*m*_0_ = 9.11 × 10^−31^ kg).

**Table 6:** Effective masses of conducting electrons and holes for Cs_2_AgCrI_6_^a^

System		Γ→L	Γ→X	Γ→L	Γ→X	Γ→L	Γ→X
Cs_2_AgCrI_6_	m_h_*/m_0_ (*up*)	−0.64	−0.52	−0.64	−0.52	−1.25	−12.96
	m_e_*/m_0_ (*up*)	0.35	0.48				

^a^m_h_* and m_e_* in in units of electron rest mass *m*_0_ (*m*_0_ = 9.11 × 10^−31^ kg).
